# Metastases in patients with adjuvant therapies: How to proceed?

**DOI:** 10.1111/jdv.70019

**Published:** 2025-10-25

**Authors:** Eftychia Chatziioannou, Martin Röcken

**Affiliations:** ^1^ Department of Dermatology University Medical Center Tübingen Germany

Originally based on experimental models in mice, two entirely different concepts of cancer therapy have been translated into clinical medicine over the past 20 years. One is based on the inhibition of ‘immune breaks’, like the interaction between ‘programmed death‐1’ (PD‐1) and its ligand, PD‐L1, primarily by monoclonal antibodies (mAbs). The other is based on the blocking of aberrantly activated tumour driver molecules with small molecular compounds, in the case of melanomas, inhibitors of BRAF^v600E^ and MEK. Both treatments were introduced into clinical practice about 10 years ago and have improved the outcome of patients with metastatic melanoma. Today, both treatments are approved as adjuvant therapy for patients at increased risk for metastatic recurrence following primary therapy of high‐risk melanomas and again are used as second adjuvant therapy after R0‐resection of metastatic recurrences.

While multiple studies document the efficacy of these therapies in metastatic and adjuvant settings, these therapies protect less than half of patients from further progression of melanoma metastases. There is therefore an important clinical need to evaluate the efficacy of immune therapies using anti‐PD‐1 mAbs and of BRAF/MEK inhibitors under these settings. The manuscript by Schumann et al.^1^ in this issue now addresses the potential use of these two types of approved therapies as secondary adjuvant therapy in patients with ROresection of metastases that had already received adjuvant therapy of stage III or IV melanomas, a frequent clinical problem that has not been explicitly investigated in randomized studies. This retrospective study may be criticized for weaknesses, but it provides valuable insights into the current situation of patients that develop surgically accessible metastases months or years after adjuvant melanoma therapy. While the comparison of independent analyses may be difficult, the real‐life data reported here show that more than 90% of the treated patients are still alive 24 months after surgery, independent of the type of therapy. In contrast, prior to the introduction of these systemic therapies, the 24‐month overall survival following metastasectomy was only about 20%–30%.^2^

The key information of the manuscript reported here^1^ results from the comparison of the recurrence‐free survival after initiation of the second adjuvant therapy (RFS2). While the overall survival data do not show a significant difference at 24 months, patients treated with BRAF/MEK inhibitors had a roughly two‐fold better RFS2 at 24 months than patients treated with anti‐PD‐1 therapies. Patients that relapsed after an adjuvant BRAF/MEK inhibitor therapy did especially poorly when they were switched to anti‐PD‐1 therapies; the opposite, switching from an adjuvant anti‐PD‐1 therapy to a secondary adjuvant therapy with BRAF/MEK inhibitors resulted in a significantly better RFS2.

The authors speculate that this difference in RFS2 may result from an unfavourable tumour immune environment, as cancer immune responses arrest cancer cells through IFN‐γ‐dependent signals.^3^ When the IFN‐γ signalling is abrogated or when cancers/melanomas have lost molecules required for the intracellular IFN‐γ signalling (Figure 1), cancer immune responses are not only entirely inefficient, they may even paradoxically enhance cancer growth.^3^, ^4^ Such data, originally again obtained from experimental tumours in mice, have more recently been investigated in RFS2 melanoma metastases from patients treated with BRAF/MEK or PD‐1 inhibition. Analyses of metastases after melanoma therapies revealed that the melanoma cells in these RFS2 metastases had new defects either in genes coding for molecules needed for appropriate IFN‐γ signalling or needed for an appropriate cellular growth arrest.^4^, ^5^ Together with the data shown here, these findings may have two important implications: they show the importance of developing predictive tests that identify those melanoma metastases that fail to respond to immune therapies and provide a basis for the development of novel therapeutic concepts.

**FIGURE 1:**
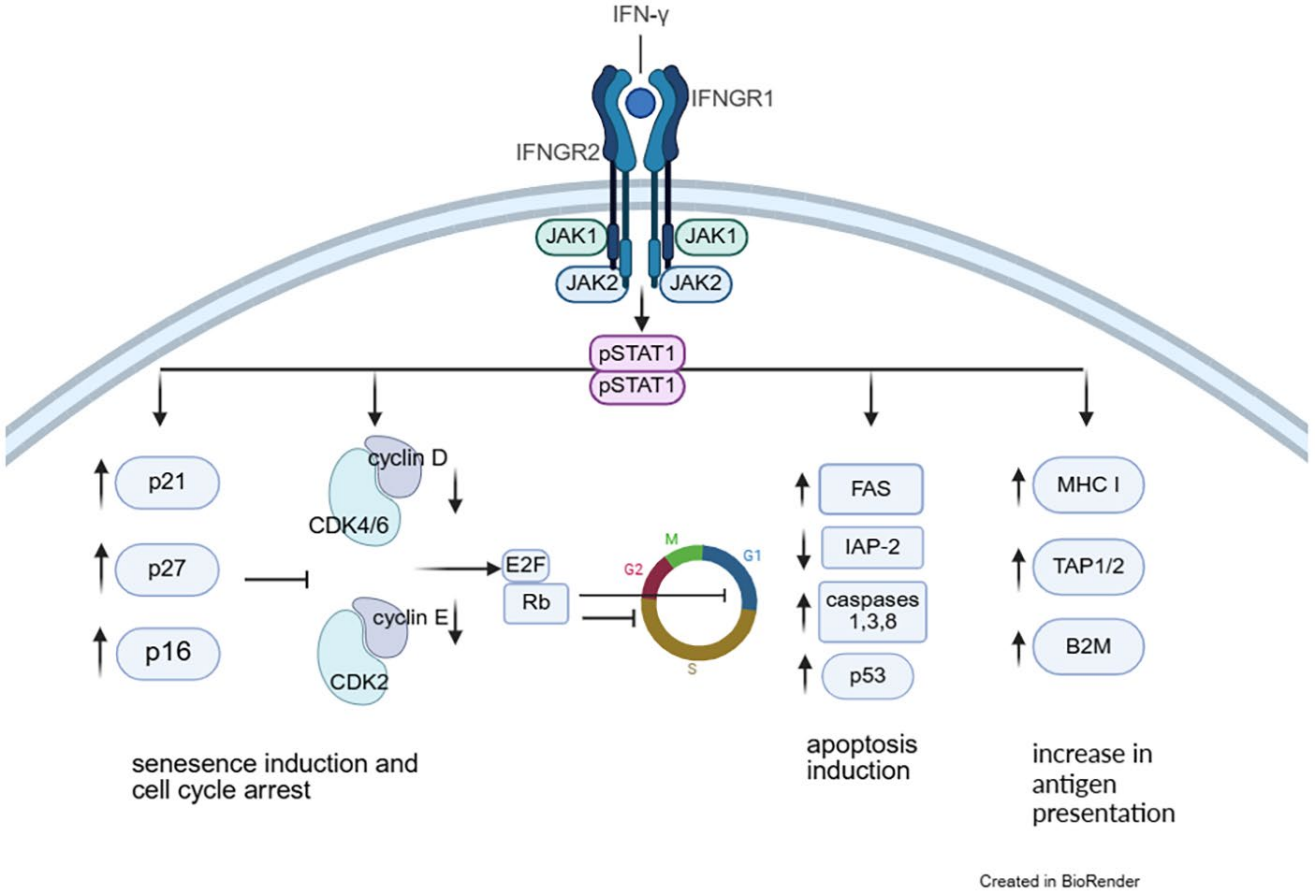
Effects of the IFN‐γ signalling on cancer immune control mechanisms that are frequently abrogated in treatment‐resistant RFS2 metastases following previous BRAF/MEK or PD‐1 inhibition.^4^, ^5^ Important pathways are loss of IFN‐γ‐dependent senescence induction/tumour growth arrest, loss of apoptosis induction, or impaired antigen presentation. These defects contribute to the resistance of primary and RFS2 metastases to anti‐PD1 immune therapies.

## CONFLICT OF INTEREST STATEMENT

Prof. Martin Röcken has received grants for studies and research projects from: Alcedis, Almirall Hermal, AstraZeneca, Biontech, Bristol‐Myers Squibb, COLDPLASMATECH, Deutsche Forschungsgemeinschaft, Deutsche Krebshilfe, Genentech, Horizon Therapeutics, HUYA Bioscience, HYPharm, Immunocore, Incyte, InflaRx, IO Biotech, IQVIA, Kartos Therapeutics, Leo Pharma, MSD Sharp & Dohme, Mühlenkreiskliniken Minden, NeraCare, Novartis Pharmaceuticals, Pfizer, Philogne, Pierre Fabre, Regeneron Pharmaceuticals, Replimune Group, RHEACELL, Roche, Sanofi Aventis, SkylineDx, Sun Pharma, Syneos Health, Takeda Pharmaceutical, Technische Universität Dresden, Unicancer, Universitätsklinikum Essen, Universitätsklinikum Freiburg, Universitätsklinikum Heidelberg and Wilhelm Sander‐Stiftung. He has received travel support from the European Academy of Dermatology and Venereology and the International League of Dermatological Societies. Dr. Eftychia Chatziioannou has no conflict of interest.

## Data Availability

Data sharing is not applicable to this article as no new data were created or analysed in this study.
